# Targeted Inactivation of Dipeptidyl Peptidase 9 Enzymatic Activity Causes Mouse Neonate Lethality

**DOI:** 10.1371/journal.pone.0078378

**Published:** 2013-11-06

**Authors:** Margaret G. Gall, Yiqian Chen, Ana Julia Vieira de Ribeiro, Hui Zhang, Charles G. Bailey, Derek S. Spielman, Denise M. T. Yu, Mark D. Gorrell

**Affiliations:** 1 Centenary Institute, Camperdown and Sydney Medical School, University of Sydney, Sydney, New South Wales, Australia; 2 Faculty of Veterinary Science, University of Sydney, Sydney, New South Wales, Australia; University of Patras, Greece

## Abstract

Dipeptidyl Peptidase (DPP) 4 and related dipeptidyl peptidases are emerging as current and potential therapeutic targets. DPP9 is an intracellular protease that is regulated by redox status and by SUMO1. DPP9 can influence antigen processing, epidermal growth factor (EGF)-mediated signaling and tumor biology. We made the first gene knock-in (gki) mouse with a serine to alanine point mutation at the DPP9 active site (S729A). Weaned heterozygote DPP9*^wt/S729A^* pups from 110 intercrosses were indistinguishable from wild-type littermates. No homozygote DPP9*^S729A/S729A^* weaned mice were detected. DPP9*^S729A/S729A^* homozygote embryos, which were morphologically indistinguishable from their wild-type littermate embryos at embryonic day (ED) 12.5 to ED 17.5, were born live but these neonates died within 8 to 24 hours of birth. All neonates suckled and contained milk spots and were of similar body weight. No gender differences were seen. No histological or DPP9 immunostaining pattern differences were seen between genotypes in embryos and neonates. Mouse embryonic fibroblasts (MEFs) from DPP9*^S729A/S729A^* ED13.5 embryos and neonate DPP9*^S729A/S729A^* mouse livers collected within 6 hours after birth had levels of DPP9 protein and DPP9-related proteases that were similar to wild-type but had less DPP9/DPP8-derived activity. These data confirmed the absence of DPP9 enzymatic activity due to the presence of the serine to alanine mutation and no compensation from related proteases. These novel findings suggest that DPP9 enzymatic activity is essential for early neonatal survival in mice.

## Introduction

The DPP4 family of enzymes gained prominence with the success of oral DPP4 inhibitors as type 2 diabetes therapeutics [1], [2]. This enzyme family is unusual because its members, DPP4, Fibroblast Activation Protein (FAP), DPP9 and DPP8, can hydrolyze the post-proline bond two residues from the N-terminus of substrates. FAP, the closest relative of DPP4, is a potential target for cancer therapy [3], [4], [5], [6]. DPP9 and its closest relative, DPP8, are primarily under study in cell biology, immunobiology and tumor biology [7]. Medicinal chemistry has generated selective inhibitors of DPP4 and of FAP but not of DPP9 and DPP8. However, some compounds inhibit DPP9 and DPP8 while not inhibiting DPP4 or FAP [8], [9].

DPP9 overexpression impairs cell adhesion [10], is pro-apoptotic [10], [11] and causes less Akt phosphorylation following EGF-stimulation [11]. The role for DPP9-mediated proteolysis in antigen presentation involves DPP9 being rate-limiting for degradation of antigenic proline-containing peptides including the tumor-related antigen RU1_34-32_ [12]. DPP9 is in B cells and both CD4+ and CD8+ lymphocytes and is upregulated by mitogen stimulation [13], [14]. Inhibiting DPP9 and DPP8 enzymatic activity dampens lymphocyte proliferation [15], [16] making DPP9 important in immunobiology. DPP9 and DPP8 enzymatic activity can protect Ewing sarcoma cells from neuropeptide Y (NPY)-driven death [17]. The potential importance of DPP9 in tumor biology has also been shown by inhibition of DPP9 and DPP8 enhancing parthenolide's anti-leukemic activity in primary acute myeloid leukemia samples and lymphoma and leukemia cell lines [18]. Moreover, an adjuvant effect triggered by inhibition of DPP9 and DPP8 appears to be a mechanism by which the compound Val-boro-Pro mediates tumor regression [19]. DPP9 binds to the oncogene and GTPase H-Ras, but the functional consequences are unclear [11]. Many cancers display upregulated DPP9 expression [13], [20], [21], [22].

While DPP4 and FAP are predominantly expressed on the cell surface, DPP9 and DPP8 are intracellular enzymes that are ubiquitously expressed in tissues and most cell lines [3], [13]. DPP9 and DPP8 are strongly expressed in lymphocytes and epithelial cells and in lymph node, thymus, spleen, liver, lung, intestine, pancreas, muscle and brain [13], [14], [23], [24].

Little is known of the natural substrates of DPP9 but it can cleave the DPP4 substrates glucagon-like peptide (GLP)-1, GLP-2, peptide YY and NPY *in vitro* and may cleave NPY inside cells [17]. Some very recently identified potential DPP9 substrates, including adenylate kinase 2 and calreticulin, suggest a potential role in energy homeostasis [25]. These data suggest that the biological roles of DPP9 *in vivo* are probably diverse such that the major role of DPP9 may be elusive.

Until now, no lethal gene knockout (gko) mouse for a protein that possesses DPP activity has existed [3]. The DPP4 gko and FAP gko mice are phenotypically healthy [13], [26], [27]. DPP4 gko mice have improved glucose tolerance after a glucose challenge and resist diet-induced obesity [27], [28]. Both DPP4 and FAP gko mice have reduced fibrosis in liver injury [29]. The knockout mice have been useful tools in evaluating the therapeutic potential of DPP4 and FAP enzyme inhibitors. However, care is necessary in evaluating such studies, as these animals have complete ablation of all protein function, rather than specific ablation of the enzymatic function alone [3], [5].

A report that certain DPP inhibitors that inhibit DPP9 and DPP8 activity are toxic *in vivo* [15] is controversial [2]. Several subsequent studies indicate that DPP9 and DPP8 [30] or DPP9, DPP4 and DPP8 [31], [32] can be inhibited in adult rodents without harm.

Structurally, DPP9 is predominantly a dimer [33] and has high sequence and topological homology with DPP4 [21], as best seen by protein structure modeling [34]. Human recombinant DPP9 and natural bovine DPP9 protein have been characterized [21], [34], [35]. Interestingly, DPP9 protease activity may be influenced by several natural processes. It is redox-responsive; in oxidizing conditions the enzymatic activity is inhibited, likely due to reversible intra-molecular disulfide bonding between cysteine residues [34]. DPP9 is acetylated [36], which might influence activity. DPP9 has a novel SUMO1-specific interacting motif that, when engaged, allosterically upregulates DPP9 enzymatic activity [37]. Such changes in DPP9 enzymatic activity would be expected to influence downstream signaling pathways.

DPP9 has an active-site catalytic triad that is conserved across the DPP4 protein family and can hydrolyze the post-proline bond two residues from the N-terminus of peptide substrates. The DPP4 family proteins are atypical serine proteases that have the catalytic triad in the reverse order to trypsin and are unusual in that the catalytic pocket is buried within the protein. In the 863 amino acid isoform (short form) of DPP9, the triad consists of Ser729, Asp807 and His839. We showed that mutation of the catalytic serine of DPP9 to alanine in amino acid position 729 of the short form of DPP9 (S729A) ablates enzymatic activity without affecting other characteristics of this protein [11], [38]. To examine the *in vivo* significance of DPP9 enzymatic activity, the first gki mouse was generated containing the S729A point mutation. This biological strategy is expected to mimic the effect of a non-toxic selective DPP9 inhibitor administered from the time of fertilization.

## Materials and Methods

### Ethics statement

The animal experiments were approved by the local Animal Ethics Committee of the University of Sydney (Ethics protocols K75/6-2012/3/5753 ‘Production of mice for studies on dipeptidyl peptidases’ and K75/5-2012/3/5754 ‘The biological roles of dipeptidyl peptidases’) and conducted in accordance with all applicable laws and guidelines.

### Generation of DPP9 enzyme-inactive mice

**Figure 1:**
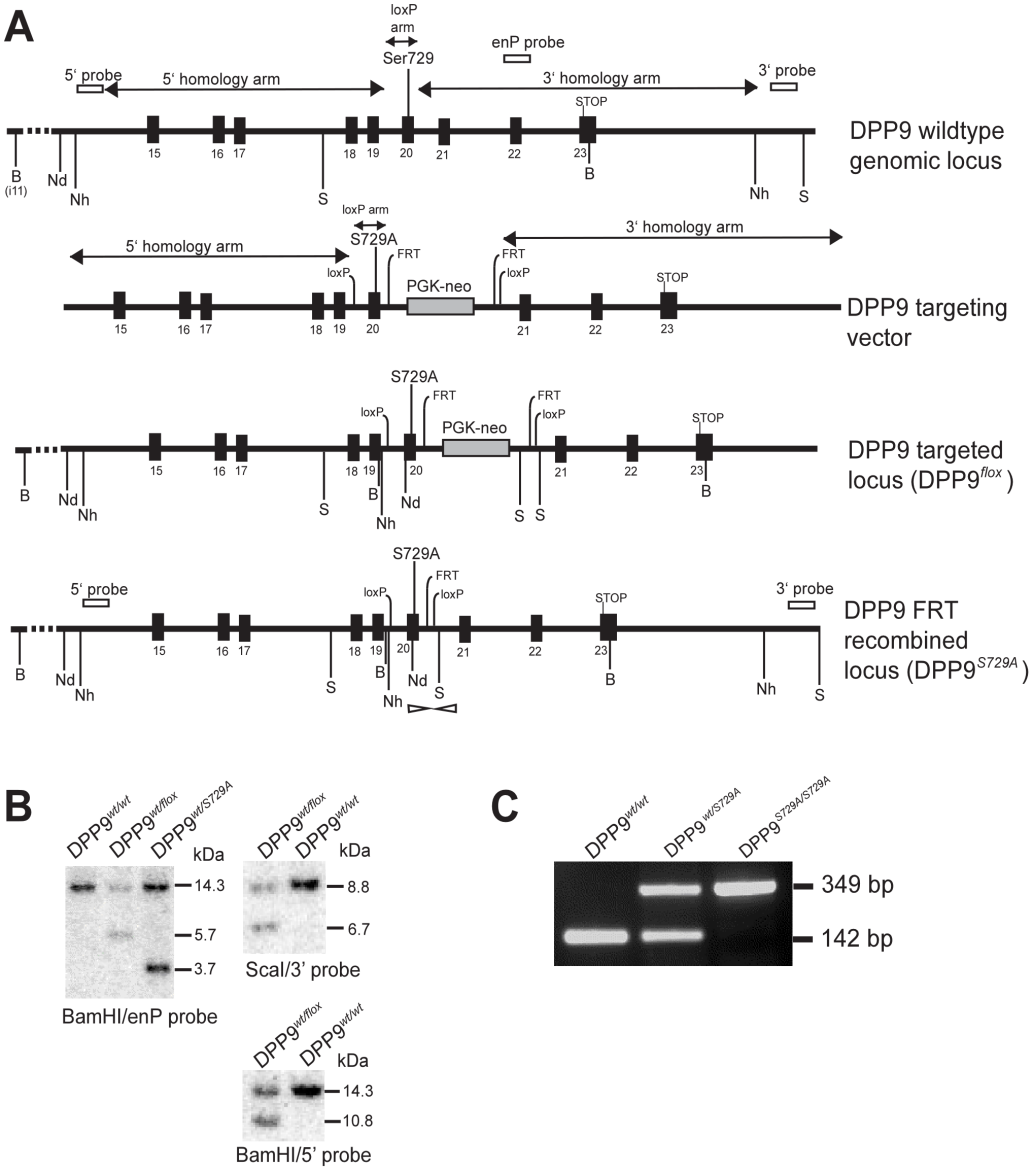
Generation of DPP9*^wt/S729A^* mice. (**A**) Representation of the DPP9 wild-type locus, targeting vector, targeted locus, and FRT recombined locus. 5′ probe, 3′ probe, enP probe, approximate PCR screening primer positions (open arrowheads) and restriction sites (Nh- NheI, S- ScaI, Nd- NdeI, B-BamHI) are indicated. (**B**) Southern blot screening confirming targeted recombination at each end of DPP9 locus: enP probe on BamHI digest (DPP9*^wt/wt^*, DPP9*^wt/flox^* and DPP9*^wt/S729A^* offspring from a wt/flox x FLPe mating), 3′ probe on ScaI digest and 5′ probe on BamHI (ES cell clone 1G6 used for microinjection). Expected sizes for wild-type and DPP9 targeted locus were detected (Table 1). (**C**) PCR screen distinguishing wild-type, DPP9*^S729A/S729A^* homozygote and DPP9*^wt/S729A^* heterozygote ED17.5 littermate embryos (wt allele, 142 bp; DPP9*^S729A^* allele, 349bp).

Generation of DPP9 enzyme-inactive mice was performed by Ozgene (Bentley, Western Australia) using standard techniques. The DPP9 targeting construct was generated by cloning polymerase chain reaction (PCR) products of the 5′ and 3′ homology arms, as well as a short loxP arm containing the S729A mutation (Figure 1A), into the plasmid backbone, FLSniper (Ozgene), which contained the Phosphoglycerate Kinase (PGK) - Neo cassette flanked by Flippase Recognition Target (FRT) sites. The S729A mutation was generated by point mutagenesis of the sequence TCC TAC to GCA TAT, which introduced an NdeI restriction enzyme site for Southern blot screening. To allow for conditional deletion of DPP9 gene expression, the same exon containing the S729A point mutation was flanked with loxP sites (exon 20 in the DPP9 short form), providing the option of exon deletion using Cre-recombinase and hence disruption of the entire protein molecule. In this study, this built-in strategy for exon excision was not utilized.

The targeting construct was electroporated into C57BL/6 Bruce 4 embryonic stem (ES) cells and clones were selected for neomycin resistance. Southern blot analyses (Figure 1B, Table 1) identified successfully targeted DPP9*^wt/flox^* ES cells. The clone chosen for generating the mutant mice, 1G6, was analysed by Southern blot and verified to be correctly targeted at each end of the construct (Figure 1B). Probing the blot with a neomycin probe confirmed a single integration event (data not shown).

**Table 1:** Southern blot strategy for screening of DPP9*^S729A^* mice.

Probe	enP probe	5′ probe	3′ probe	5′ probe	5′ probe
**Restriction enzyme**	BamHI	BamHI	Sca I	Nhe I	Nde I *
**DPP9** ***^wt^*** ** allele (kb)**	14.3	14.3	8.8	13.6	23.0
**DPP9** ***^flox^*** ** allele (kb)**	5.7	10.8	6.7	7.5	9.4
**DPP9** ***^S729A^*** ** allele (kb)**	3.7	10.8	6.7	7.5	9.4

Southern blot analyses identified successfully targeted DPP9*^wt/flox^* embryonic stem cells using probes described in this table. *NdeI restriction site was inserted during introduction of DPP9*^S729A^* mutation.

The DPP9*^wt/flox^* ES clone was microinjected into BALB/c blastocysts which were implanted into pseudo-pregnant foster mothers. Chimeric male mice from the resultant litters were mated with C57BL/6 wild-type females and offspring with coat-colour transmission were genotyped for germline transmission of the S729A mutation by Southern blot of tail DNA (Figure 1B). Genotyping confirmed germline transmission of the DPP9 serine-to-alanine point mutation locus.

The neo cassette was FLPe-deleted *in vivo* using the FRT sites by mating heterozygous DPP9^w*t/flox*^ mutant males with transgenic female wt/FLPe mice expressing the enzyme Flp recombinase. Southern blot genotyping of offspring confirmed deletion of the PGK-neo selection cassette. Resultant positive offspring (DPP9*^wt/S729A^*) were mated with C57BL/6 mice for removal of the FLPe gene.

Routinely, DPP9*^wt/S729A^* intercross progeny were genotyped by Southern Blot using the 5′ or 3′ probes (Figs. 1A and 1B, Table 1), or by PCR using primers (Figure 1C, Table 2) and Advantage2 Taq Polymerase (Clontech, Mountain View, CA). The PCR cycles used were 94°C for 2 min, then 94°C for 30 sec, 60°C for 30 sec, 68°C for 30 sec repeated 39 times followed by 68°C for 10 min and 10°C on hold. Sequencing of gel-isolated PCR product confirmed the correct amplification of the mutant allele region and the presence of the S729A mutation in the 349 bp PCR fragment.

**Table 2:** PCR primers for routine screening of DPP9*^S729A^* mice/ embryos/ MEFs.

Primer name	mDPP9_E20.F	mDPP9_i20.R
**Sequence (5′ - 3′)**	AAGTATGGCTTCATTGACTTGAGC	GGTGGGCATCAGGCTGCAGGTGG
**Wild-type allele (bp)**	142	
**DPP9** ***^S729A^*** ** allele (bp)**	349	
**Annealing temp. (°C)**	64	

DPP9*^wt/S729A^* intercross progeny and MEFs generated from these progeny were genotyped by PCR using primers and PCR cycle outlined below.

### Embryonic and early neonate studies

Embryonic day (ED) 12.5 (ED12.5), ED13.5, ED15.5 and ED17.5 embryos were harvested from DPP9*^wt/S729A^* intercrosses. Umbilical cord or embryonic tail was proteinase K- treated for genotyping, while the rest of the embryo was formalin fixed overnight.

For post-natal studies, both observational and experimental studies were undertaken to define the phenotype. In observational studies, hourly litter examinations from birth ensured retrieval of non-surviving pups for genotyping and formalin fixation. All pups were closely observed for physical activity, breathing difficulties and unusual behaviours or appearance. In litters used for experimental studies, all pups were euthanized within six hours of birth, tails retained for genotyping and head and body fixed in 10% neutral buffered formalin.

### Histochemical studies

To undertake histological observations, paraffin-embedded pups were sectioned at 5 µm and H&E stained. To immuno-localise DPP9 protein, the anti-DPP9 antibody (Abcam; Cambridge, UK, #ab42078, 1∶100) was used which bound to both the wild-type and S729A DPP9 proteins. Briefly, 5 µm sections were deparaffinized and rehydrated and then a pressure cooker and Universal Decloaker solution (Biocare Medical, Concord, CA) was used to retrieve antigen. Sections were then covered with Background Sniper (Biocare Medical) for 10 mins, rinsed in PBS and incubated at room temperature for 1 h with primary antibody with 1% BSA in PBS and Renaissance Background Reducing Diluent (Biocare Medical). After thorough washing in PBS, sections were incubated for 30 mins with goat anti-rabbit conjugated to HRP (Dako, Glostrup, Denmark, #P0448, 1∶100), washed and then stained in 3,3-diaminobenzidine (DAB) with H_2_O_2_. Bright-field imaging was performed using a Leica DM6000B microscope.

### Immunofluorescence Imaging

DPP9-WT-EGFP and enzyme-inactive mutant DPP9-S729A-EGFP constructs [10] were transiently transfected into human hepatocarcinoma cell line (Huh7) cells using Lipofectamine® 2000 (Invitrogen, Carlsbad, CA, USA) at 0.4 µg/µL. Forty hours post-transfection, cells were fixed with 4% paraformaldehyde as described previously [10]. Immunostaining used rabbit anti-DPP9 antibody (Abcam, #ab42080, 1∶200), Alexa Fluor® 647 goat anti-rabbit IgG (Invitrogen, #A-21245, 1∶200) and DAPI counterstain. Z-stack images were captured on a Leica TCS SP5 confocal microscope and the stacks above and below the nucleus were removed. The remaining stacks were reconstructed into a z-projection with ImageJ software.

### SDS-PAGE urine analysis

In litters used for detection of protein in urine, pups were euthanized within 6 hours of birth by decapitation, then urine collected and analyzed by a method modified from Putaala *et al* [39]. Briefly, 3 µL of urine from each pup together with 1 µL of SDS-sample buffer was run on a 4-12% Bis-Tris SDS-PAGE gel (Invitrogen) under non-reducing conditions. Gels were stained with Coomassie Blue.

### Generation and immortalization of mouse embryonic fibroblasts (MEFs)

ED13.5 embryos from DPP9*^wt/S729A^* intercrosses were harvested under sterile conditions and MEFs prepared using standard protocols [40]. Briefly, the uterus of each pregnant mouse was removed post mortem and each embryo separated from its placenta and surrounding membranes and placed in complete growth medium (DMEM with 10% FCS). The embryonic liver and head were removed and then the body was homogenized in TrypLE (Life Technologies, Mulgrave, Victoria, Australia) using a syringe and 23 G needle. The homogenate was incubated at 37°C for 10 min before transfer to complete growth medium and culturing at 37°C with 5% CO_2_ as passage zero.

Genotyping by PCR was performed on DNA isolated from embryonic head treated with proteinase K using the Wizard® Genomic DNA isolation kit (Promega, Madison, WI, USA).

After the third passage, primary MEFs from wild-type and DPP9*^S729A/S729A^* littermates were infected with SV40 large T antigen-expressing lentivirus to generate immortal cell lines [41]. Briefly, primary MEFs were transduced with the pFU-SV40-LT-puro lentiviral vector encoding the SV40 large T antigen pseudotyped with the Moloney murine leukemia virus ecotropic envelope (pCAG4-Eco) and the structural component (pCMV ð R8.2). Cells (1×10^5^) plated out 24 h earlier were transduced in 6-well culture plates containing 2 mL complete growth medium and 8 µg/mL Polybrene (Sigma-Aldrich, Castle Hill, Australia), ‘spinoculated’ at 1,500 rpm for 1 h and then cultured for 4 days at 37°C. Puromycin selection (1 µg/mL) was added for 3 days to remove non-transduced cells. Colonies of immortalized MEFs emerged after 10 days.

### Enzyme assays

DPP8/9 enzyme assay using the DPP fluorogenic substrate H-Gly-Pro-AMC (Mimotopes, Clayton, Victoria, Australia) was adapted from described methods [13], [42]. Briefly, 3×10^4^ MEF cells or 10 µg of liver sample lysate per well were added to black 96-well plates (Greiner Bio One, Frickenhausen, Germany) in triplicate in the presence or absence of a selective DPP4 inhibitor, sitagliptin (Merck, Rahway, NJ, USA), at 1 µM and TE buffer to a volume of 50 µL per well. 50 µL of substrate was added to a final reaction concentration of 1 mM in TE buffer (pH 7.6) with 5% methanol. The fluorescence produced by substrate cleavage was monitored every 5 min over 1 h at 37°C in a Polarstar Omega microplate reader (BMG Labtech, Offenburg, Germany) with excitation at 355 nm and emission at 450 nm.

### Immunoblotting

For immunoblot sample preparation, MEFs were harvested by trypsinization and washed with ice-cold PBS before re-suspension in ice-cold lysis buffer (50 mM Tris-HCl, 1 mM EDTA, 1 mM MgCl_2_, 150 mM NaCl, 1% Triton-114, 10% glycerol, 1× Roche complete protease inhibitor cocktail (Roche Applied Science, Indianapolis, IN, USA; pH 7.6) and stored at −20°C. Frozen neonate mouse liver samples were homogenized in lysis buffer using a bead-based homogenizer (TissueLyser, Qiagen Venlo, Netherlands) at 4°C. Protein concentration was determined using the Micro BCA Protein Assay Kit (Thermo Scientific, Waltham, Massachusetts, USA) following the manufacturer's protocol.

Whole cell lysates (50 µg protein per track) were resolved on 4–12% Bis-Tris SDS-PAGE (Invitrogen) followed by immunoblotting with anti-DPP9 antibody (1∶2000) [11] and anti-β-Actin (Sigma-Aldrich, #A2103, 1∶5000). Relative band intensities were quantitatively analyzed using ImageJ and normalized against control proteins as indicated.

### Quantitative real-time PCR (qPCR)

Total RNA was isolated using Trizol (Invitrogen) and cDNA synthesis was performed using SuperScript VILO cDNA Synthesis Kit (Invitrogen). qPCR was performed as described previously [14] using Taqman gene expression assays (Applied Biosystems, Foster City, CA, USA) for mouse DPP4 (Mm00494548_m1), DPP8 (Mm00547049_m1), DPP9 (Mm00841122_m1) and FAP (Mm00484254_m1), with β-Actin (Mm00607939) as a standard.

## Results

### DPP9*^S729A/S729A^* homozygotes die in the neonatal period

Heterozygous DPP9*^wt/S729A^* mice on a C57BL/6 background were generated and sequencing of purified PCR products verified the presence of the S729A mutation in the DPP9 allele (Figure 1). However, intercrossing of DPP9*^wt/S729A^* mice and subsequent absence of any detectable DPP9*^S729A/S729A^* homozygote offspring at weaning was suggestive of lethality.

To determine the time of death, litters were observed and genotyped at embryonic and early neonatal stages. Live homozygote embryos and pups were detected in ED 12.5, 13.5, 15.5, 17.5 and early neonate (Day 1) litters. Analysis of embryo genotype numbers (n = 41) confirmed that the DPP9 S729A allele was present in the expected Mendelian frequency (data not shown). Thus, it appeared that enzyme-active DPP9 is not essential for development of the mouse embryo to term.

Directly after birth, normal cyanosis was present and all pups breathed normally and became pink (n = 92). Littermates were of a similar size and weight with no significant gender or genotype difference found for Day 1 neonates (*p*>0.05) (Table 3). All appeared to feed normally with milk in the stomach shown by a milk spot and later by histological examination of whole neonate sections. At this early stage, pups showed no discernible behavioral differences, however, after several hours, some pups were found dead or showing weak respiratory movements and lethargy. There were no visual differences between genotypes in skin color or hydration.

**Table 3:** Average neonate weights (in grams) for genotype and gender on Day 1 after birth.

	Genotype				Gender		
	DPP9*^S729A/S729A^*	DPP9*^wt/S729A^*	DPP9*^wt/wt^*	Total	♂	♀	Total
Neonate number	21	53	18	92	40	52	92
Observed average weight	1.09±0.13	1.12±0.13	1.16±0.11		1.13±0.13	1.09±0.12	
Expected average weight	1.12	1.12	1.12		1.12	1.12	
	***χ^2^ = 0.002*** ** ***p = 0.999***				***χ^2^ = 0.003 p = 0.959***		

Observed weights are expressed as the mean ± standard deviation.

Investigating the mortality of newborn pups revealed that, of the 27 found dead in the first 24 hours, all were either DPP9*^S729A/S729A^* or DPP9*^wt/S729A^*. This suggested a negative effect on survival to weaning for offspring carrying the S729A mutation.

Subsequent genotyping of newborn DPP9*^wt/S729A^* intercross pups showed that the DPP9 S729A allele occurred in the expected Mendelian ratios. From 33 litters from DPP9*^wt/S729A^* breeding pairs, 317 pups were produced with 68 DPP9*^S729A/S729A^*, 167 DPP9*^wt/S729A^* and 82 DPP9*^wt/wt^*. Chi-square analysis confirmed no significant deviation from a 1∶2∶1 ratio (*p*>0.05). Similarly, a lack of significant gender bias shown in neonates precludes a gender effect by the DPP9 S729A allele on embryonic development (Table 4).

**Table 4:** Neonate mouse genotype and gender numbers and ratios compared to expected Mendelian ratios.

	Genotype				Gender		
	DPP9*^S729A/S729A^*	DPP9*^wt/S729A^*	DPP9*^wt/wt^*	Total	♂	♀	Total
Observed number	68	167	82	317	123	147	270
Expected number	79.25	158.5	79.25		135	135	
Expected ratio	0.25	0.5	0.25		0.5	0.5	
Actual ratio	0.21	0.53	0.26		0.46	0.54	
	***χ^2^ = 2.148 p = 0.342***				***χ^2^ = 2.133 p = 0.144***		

At weaning, from 110 tracked DPP9*^wt/S729A^* intercross litters resulting in 563 weaned pups, only DPP9*^wt/S729A^* and DPP9*^wt/wt^* pups were present with no DPP9*^S729A/S729A^* pups. Interestingly, Chi-square analysis showed a significant bias towards DPP9*^wt/wt^* pups (*p* = 0.008), rather than an expected 2∶1 genotype ratio (Table 5). This confirmed a heterozygous effect on survival to weaning. The group of surviving DPP9*^wt/S729A^* heterozygous pups also showed a slight variation from a 1∶1 ratio of males to females (Table 5), however this result is consistent with previous studies on mouse sex ratios [43], [44], [45] and, thus, not attributable to the heterozygous effect. There was no gender bias amongst pups that did not survive to weaning.

**Table 5:** Weaned mouse genotype and gender numbers and ratios compared to expected Mendelian ratios.

	Genotype			Gender		
	DPP9*^wt/S729A^*	DPP9*^wt/wt^*	Total	♂	♀	Total
Observed number	346	217	563	307	256	563
Expected number	375.5	187.5		281.5	281.5	
Expected ratio	0.67	0.33		0.50	0.50	
Actual ratio	0.62	0.38		0.55	0.45	
	***χ^2^ = 6.969 p = 0.008***			***χ^2^ = 4.620 p = 0.032***		

Therefore, while the DPP9*^S729A/S729A^* embryos and neonates were indistinguishable morphologically from their wild-type and heterozygote littermates, neonates died shortly after birth indicating that DPP9 enzymatic activity is required for neonatal survival.

### Early neonate DPP9*^S729A/S729A^* homozygotes show no histological, histochemical or urinary protein difference from their heterozygous and wild-type littermates

**Figure 2:**
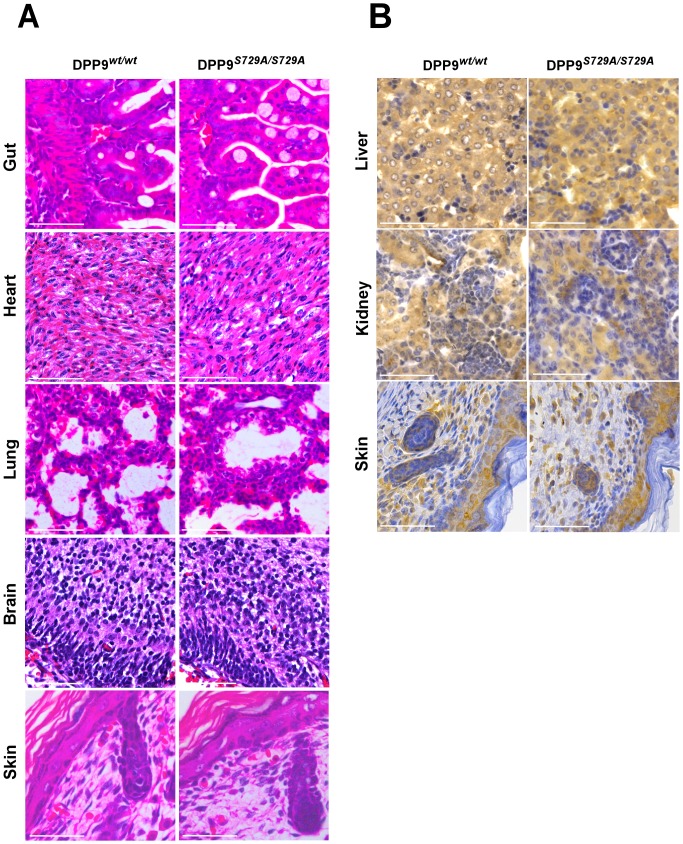
Histology and immunohistochemistry of early neonate tissues are comparable between genotypes. (**A**) Light micrographs of H&E-stained DPP9*^S729A/S729A^* lung, heart, gut, brain and skin tissue compared to DPP9*^wt/wt^* show no differences in histological structure and (**B**) Anti-DPP9 antibody staining showed no DPP9 localization differences between DPP9*^wt/wt^* and DPP9^S*729A/S729A*^ early neonate sections. Scale bar 50 µm.

Light microscopic observations of H&E-stained whole neonate sections (Day 1) by a trained pathologist (DSS) revealed no histological differences between DPP9^S*729A/S729A*^ pups (n = 4) and their DPP9*^wt/S729A^* (n = 2) and DPP9*^wt/wt^* (n = 4) littermates. Organs examined were gut, heart, lung, brain and skin (Figure 2A) along with kidney, liver, pancreas, thymus, cartilage and muscle (not shown).

Immunohistochemistry using an antibody specific to DPP9 [24] that has been used extensively by us [11], [13], [14] and others [24], [46], showed no DPP9 localization differences between DPP9*^wt/wt^*, DPP9*^wt/S729A^* or DPP9^S*729A/S729A*^ on early whole neonate sections. Many neonate organs were strongly immuno-positive for DPP9 protein, most notably kidney, skin, liver (Figure 2B), brain and thymus. Overall, the immunostaining was consistent with published data [13], [23].

Neonatal death of mice within 24 hours of birth can be due to nephrin deficiency and, hence, massive proteinuria [39]. SDS-PAGE analysis of urinary protein in early neonates (data not shown) showed no proteinuria and no urinary protein band differences between DPP9*^wt/wt^*, DPP9*^wt/S729A^* or DPP9^S*729A/S729A*^ littermates, thus excluding renal damage as a cause of death.

### DPP9*^S729A/S729A^* mouse embryonic fibroblasts express intact DPP9 mRNA and enzyme-inactive DPP9 protein

To verify the expression of DPP9 enzyme-inactive protein, immortalized MEFs and neonate livers of *DPP9^S729A/S729A^* and wild-type littermates were characterized for DPP mRNA and protein expression and activity. DPP9 protein and mRNA levels were measured by Western blot and qPCR respectively.

**Figure 3:**
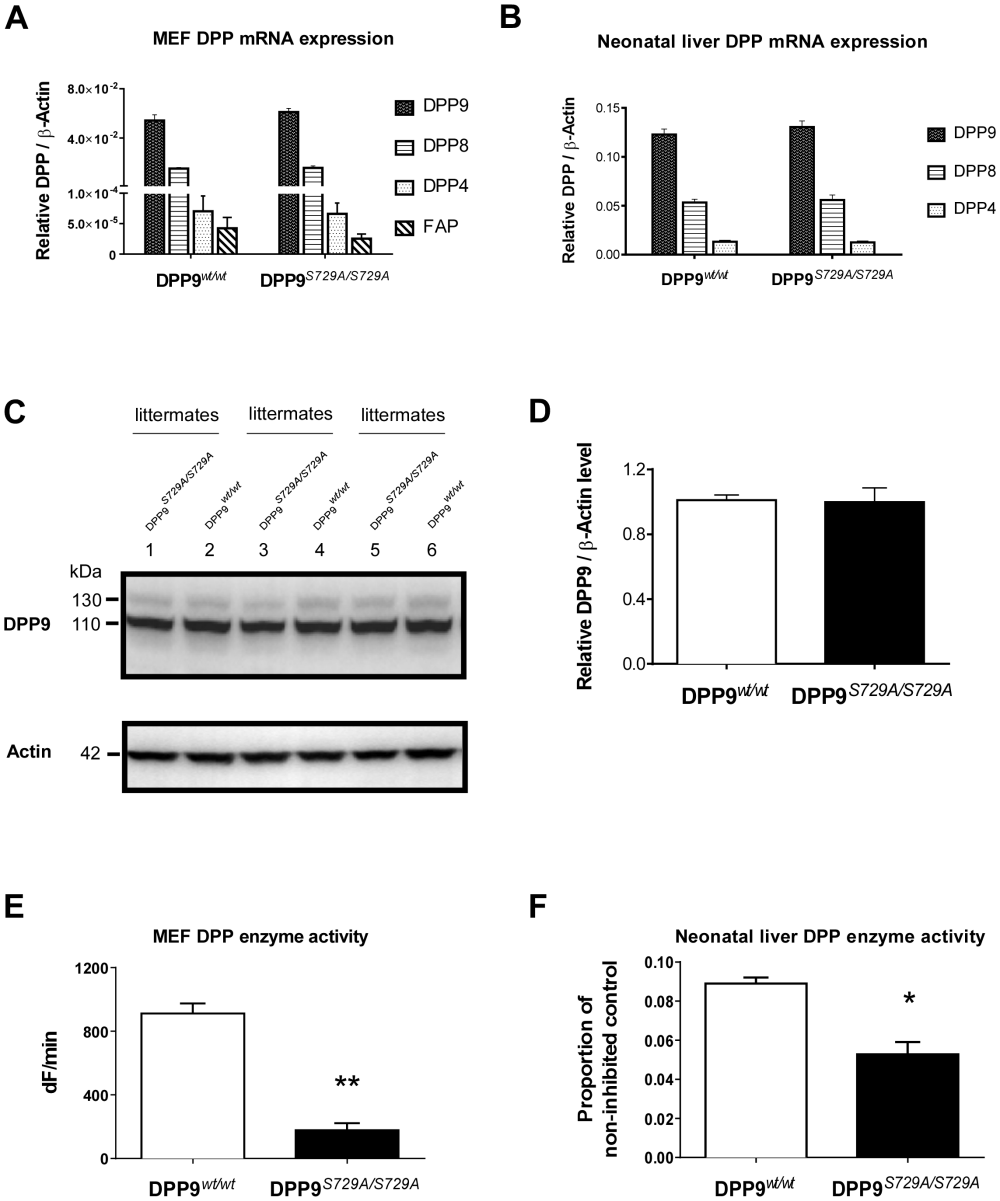
DPP9 expression in DPP9*^wt/wt^* and DPP9*^S729A/S729A^* MEFs and neonatal liver is equivalent. By qPCR, (**A**) DPP9*^S729A/S729A^* MEFs (n = 3) and (**B**) neonatal liver (n = 8) showed similar mRNA expression levels of DPP4, DPP8, DPP9 and FAP compared to DPP9*^wt/wt^*. (**C**) Similar levels of intact DPP9 protein were detected by Western blotting in DPP9*^S729A/S729A^* MEFs compared to DPP9*^wt/wt^* littermate MEFs. (**D**) Densitometry analysis of this Western blot; the 110 kDa and 130 kDa bands were combined. DPP8/9 enzyme assays showed less enzymatic activity in (**E**) DPP9*^S729A/S729A^* MEFs compared to WT MEFs (n = 3), **p<0.001 and (**F**) DPP9*^S729A/S729A^* neonatal livers (n = 4) compared to WT livers (n = 6), *p<0.05. The DPP enzyme activity contributed by DPP9 and DPP8 is the proportion of non-inhibited control activity which was calculated as the hydrolysis of H-Gly-Pro-AMC observed after inhibition of DPP4 by sitagliptin. dF is change in fluorescence.

qPCR results indicated that *DPP9^S729A/S729A^* MEFs and neonatal liver expressed similar levels of DPP9 mRNA, as well as FAP, DPP8 and DPP4 mRNA, to wild-type (Figs. 3A and 3B). When immunoblotting with a DPP9 antibody, DPP9 bands run at different sizes in different cell lines [11], [14]. Using this antibody with MEFs showed similar levels of intact DPP9 protein expression in *DPP9^S729A/S729A^* MEFs and wild-type MEFs (110 and 130 kDa) (Figure 3C) and confirmed by densitometry (Figure 3D). These results demonstrated that the targeted point mutation did not influence the expression of DPP9 mRNA and protein. In addition, it indicates that there was no detectable compensatory up-regulation of the other DPPs due to the lack of DPP9 enzymatic activity.

Enzyme activity derived from DPP9 and DPP8 can be measured in the presence of a DPP4 inhibitor and a substrate that is hydrolyzed by all three proteases [13]. Enzyme activity assays on whole MEFs showed that *DPP9^S729A/S729A^* MEFs had significantly less DPP enzymatic activity than wild-type MEFs (Figure 3E). Moreover, in neonatal liver lysates (Figure 3F), *DPP9^S729A/S729A^* neonatal liver contained significantly less DPP8/9-derived enzymatic activity than wild-type liver, which is consistent with a lack or large reduction of DPP9 enzymatic activity. Together with the Western blot and qPCR data, this provides evidence that DPP9-S729A enzyme-inactive protein was expressed at similar levels to DPP9-WT enzyme-active protein in MEF cell lines and neonatal livers.

### DPP9-S729A and wild-type DPP9 have the same subcellular localization

**Figure 4:**
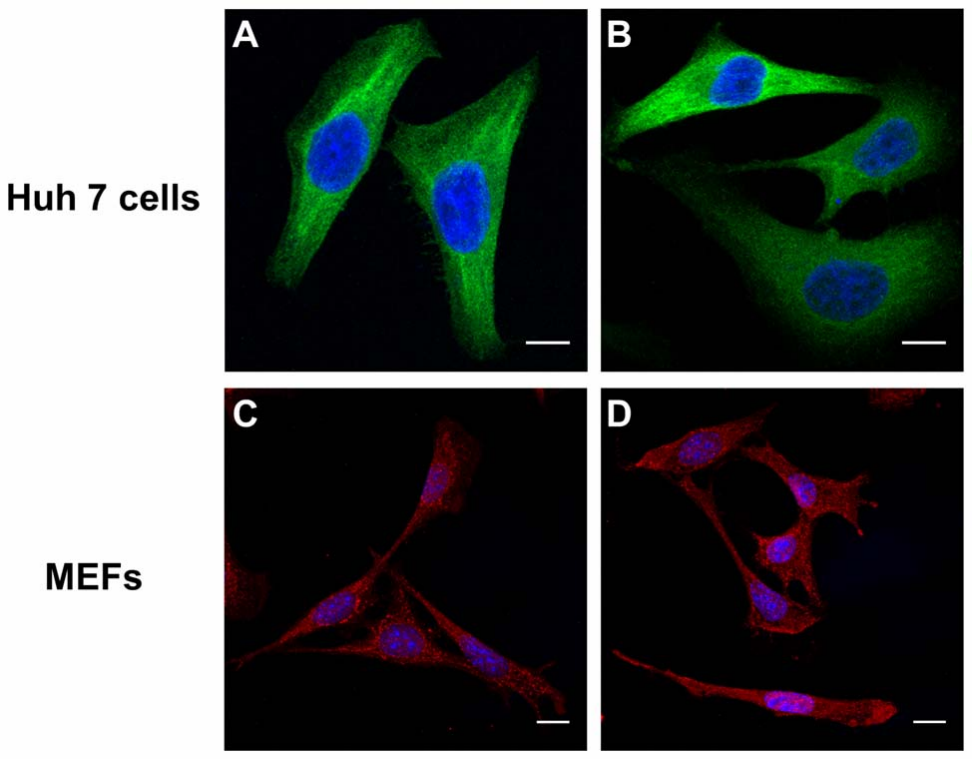
Active DPP9-WT and enzyme-inactive DPP9-S729A show the same intracellular localization. DPP9-EGFP visualization in Huh7 cells transfected with (**A**) DPP9-WT-EGFP or (**B**) DPP9-S729A-EGFP showed no localization difference. Similar expression patterns of antibody-stained DPP9 (red) were observed in (**C**) wild-type MEFs and (**D**) *DPP9^S729A/S729A^* MEFs. Confocal images were created only from z-stacks that contained the nuclear region. Scale bar 15 µm.

A point mutation in rat DPP4 [47] causes mis-localization and intracellular degradation of DPP4 rather than normal cell surface expression. To examine whether enzyme-inactive DPP9 protein and active DPP9 protein are similarly localized *in vitro*, DPP9-overexpressing Huh7 cells and endogenous DPP9 in MEFs were examined by immunofluorescence staining and confocal microscopy. DPP9-S729A-EGFP had an intracellular distribution similar to DPP9-WT-EGFP (Figure 4A). Similarly, endogenous DPP9 expression in DPP9*^S729A/S729A^* MEFs was indistinguishable from DPP9 protein in wild-type MEFs (Figure 4B).

## Discussion

In this study, we found that DPP9 enzymatic activity is essential for neonatal survival in the mouse. The mice express intact DPP9 protein but with the loss of enzymatic activity. This did not alter pre-natal development but resulted in neonatal deaths. Thus, the complete disruption of DPP9 expression was not necessary for early lethality, highlighting the biological importance of the enzyme activity of DPP9 at this stage of development. The lethality of lacking DPP9 activity indicates that DPP9 is unique such that no other enzyme can compensate for this enzyme at this developmental stage. The cause of death was not apparent. All neonates suckled and contained milk spots and males and females of each genotype were of similar body weight. No differences were seen by histological examination and no urinary proteinuria was observed.

The close structural similarity of DPP9 to DPP8 makes the development of selective DPP9 inhibitors challenging [8], [48] and has not been achieved. Thus, the DPP9 enzyme-inactive mouse is a useful and unique model for emulating the biological effects of selective DPP9 inhibition from the embryonic stage and avoids potential off-target effects that chemical compounds may produce in mice. Our genetic targeting approach is the only DPP9-specific model for understanding the biological significance of DPP9 enzymatic activity *in vivo*. The biology in this model is very different to previous studies that have treated adult animals with inhibitors of both DPP9 and DPP8 or of DPP9, DPP4 and DPP8 together [30], [31], [32].

To verify the status of DPP9 protein in our mice, the MEF and neonatal liver data indicate that the S729A mutation produced a full-length DPP9 protein with no enzymatic activity. Comparable sizes and intracellular localizations of DPP9 intact protein and similar DPP mRNA levels were shown in DPP9 enzyme-inactive MEFs compared to MEFs and neonatal liver derived from wild-type littermates. In addition, related dipeptidyl peptidases were not upregulated in the DPP9 enzyme-inactive mouse to compensate for the absence of DPP9 activity. We similarly observed in our enzyme distribution studies on DPP4 gko mice [13] and FAP gko MEFs (Hamson, Yu, Gorrell, unpublished data) that DPP4-related dipeptidyl peptidases are not upregulated in those mouse strains.

Like DPP9, DPP8 has intracellular expression [10], [49] and DPP4, FAP and DPP8 all have DPP enzymatic activity [2], [13], [21], [49], [50], [51], [52], [53]. Since DPP4 gko and FAP gko mice are phenotypically healthy, DPP9 neonate lethality was unexpected. This lethality shows that DPP4, DPP8 and FAP are unable to fulfill an essential role of DPP9 in early mouse neonate life, thereby highlighting a unique role for DPP9 activity within the DPP4 gene family.

Many factors can contribute to neonate deaths but the time of death can provide clues to the potential causes [54]. While there are numerous examples of neonatal lethality in genetically modified mice [54], very few die so quickly and without morphological differences from their heterozygous or wild-type littermates. Having survived the stress of parturition, neonates fulfill new metabolic needs by their own homeostasis, failure of which can result in poorer survival. As the DPP9*^S729A/S729A^* pups survived parturition with no obvious gross anatomical and histological differences, it seems the resultant lethality in DPP9*^S729A/S729A^* mice is probably attributable to metabolic impairment. The literature reports dysfunctions in homeostasis due to failures in autophagy [55], kidney filtration [39], transcriptional controls in cell nuclei [56] and *in vivo* glucose homeostasis [57]. The nature of the dysfunction caused by lacking DPP9 enzymatic activity at birth needs to be elucidated by further investigations.
